# Interpreting the Choice Logic Surrounding High-Scoring Students’ Enrollment in China’s Vocational Secondary–Undergraduate Articulation Program: A Theoretical Thematic Analysis of Public Discourse

**DOI:** 10.3390/bs16050734

**Published:** 2026-05-09

**Authors:** Lihua Xie, Yukun Wang, Shiyang Zeng

**Affiliations:** Institute of Vocational Education, Tongji University, Shanghai 200092, China; 2330028@tongji.edu.cn (Y.W.); 2430041@tongji.edu.cn (S.Z.)

**Keywords:** VET in China, vocational secondary–undergraduate articulation, high-scoring junior secondary graduates, rational choice theory, educational choice motivation, vocational pathways

## Abstract

In China’s increasingly stratified education system, a growing number of high-scoring junior secondary students are choosing the Vocational Secondary–Undergraduate Articulation Program over key academic senior high schools, challenging conventional assumptions about merit, school choice, and vocational education. Existing studies have mainly examined this pathway from institutional and policy perspectives, with limited attention to the micro-level decision-making logic of students and families. Drawing on rational choice theory and a theoretical thematic analysis of online public discourse, this study explores how high-scoring students and their families interpret, evaluate, and justify this educational choice. The findings show that participation in the articulation program is organized around four interrelated mechanisms: action foundations based on academic strengths and family evaluation; action purposes aimed at securing a relatively stable route to a bachelor’s degree while gaining vocational advantages; action consequences involving the weighing of risks and expected returns; and institutional and cultural impacts produced by the interaction between policy incentives and persistent academic hierarchies. The study argues that choosing this pathway is not a deviant decision, but a rational response to educational competition and structural constraints, and it sheds light on the changing legitimacy of vocational pathways in contemporary China.

## 1. Introduction

Across different countries and education systems, students’ educational choices are not merely passive responses to the perceived risks, returns, and developmental prospects associated with different pathways. Educational decision-making itself constitutes an important source of divergence in individual educational pathways (Guo & Qu, 2022). As the primary investors in their children’s education, parents shape their children’s learning goals and post-graduation choices through their own expectations (Albert et al., 2020). In China, families generally maintain strong aspirations for higher education, and high parental expectations and strict behavioral supervision are often understood as expressions of emotional warmth and parental responsibility rather than as purely controlling practices (Zhang et al., 2026; Hou, 2025). Students tend to internalize these parental expectations into their own self-expectations, while also experiencing substantial academic pressure; in this context, academic achievement is often seen not merely as an indicator of individual competence, but also as a responsibility toward parents and other significant others (Aizizi, 2026; Zhao et al., 2009; Tao & Hong, 2014). Although realistic parental educational expectations may contribute positively to adolescents’ subjective well-being, this positive effect is significantly weakened when adolescents perceive high levels of academic pressure (Lu et al., 2021).

China’s upper-secondary system is now highly expanded but remains internally stratified. In 2024, the gross enrolment rate at the upper-secondary level reached 92.0%; ordinary academic high schools admitted 10.362 million students, compared with 4.1833 million in secondary vocational education, indicating a general-vocational distribution of roughly 71.2% to 28.8% (Ministry of Education of the People’s Republic of China, 2025). At the same time, access to upper-secondary education remains socially and spatially differentiated, as community socioeconomic context continues to shape students’ chances of entering high school, especially in disadvantaged areas (Lei, 2022; Y. Wu & KC, 2023).

China’s education system has been described as a “paradigmatic case of meritocratic practice,” in which examination scores are decisive and an exam-oriented, ranking-based structure reinforces this logic (Jin & Ball, 2020; Xie & Liu, 2025). Academic performance has long been regarded as the central criterion for evaluating students and remains closely associated with access to educational opportunities and prospects for upward social mobility (Sheng, 2017). Only those who perform exceptionally well academically are considered worthy of recognition and reward, both materially and symbolically (Jin & Ball, 2020). Within this institutional and cultural context, high-scoring students have long been seen as the “winners” of academic competition and the natural candidates for elite academic schools. Here, the term “high-scoring students” does not refer to the very highest achievers in the overall secondary school hierarchy. Rather, it refers to students with relatively strong entrance examination performance within the vocational education context, many of whom would also qualify for regular, and sometimes relatively selective, general upper-secondary options.

Yet corresponding to the social expectation that high-scoring students ought to follow the academic track, vocational education has long occupied a comparatively low-status position in China. Although status differences between academic and vocational education exist in many countries, this hierarchy is especially deeply entrenched in China (Stewart, 2015). Under the enduring influence of the traditional cultural ideal that “those who excel in learning should enter officialdom,” academic achievement continues to serve as a key marker of social prestige, whereas technical and vocational education has not been granted comparable cultural legitimacy (Guan & Blair, 2024). As a result, the general academic pathway is usually constructed as the more legitimate and respectable “right path,” and is by default associated with access to higher education and a better future (Sheng, 2017). By contrast, the vocational pathway is often viewed as a compensatory option reserved for low-scoring students, a choice made only out of necessity. The relatively low social standing of vocational education and its limited opportunities for upward educational mobility can negatively affect students’ subjective social status (Yi et al., 2018).

It is precisely within this institutional, cultural, and psychological context that the recent phenomenon of high-scoring students voluntarily choosing vocational education, especially the Vocational Secondary–Undergraduate Articulation Program (“Zhongben Guantong”, hereafter the articulation program), appears both empirically unexpected and theoretically significant. This program allows students to enter a three-year vocational school and subsequently articulate into an aligned undergraduate program upon passing standardized transition assessments. Those who successfully complete the full cycle receive a bachelor’s degree and corresponding diploma, formally equivalent to those obtained through the conventional academic track (L. Liu, 2023). Once considered a marginal or alternative route, the articulation program has rapidly gained visibility, especially in metropolitan regions. In Shanghai, approximately 24,000 students applied for articulation programs in 2024, about 7000 over the previous year, and admission thresholds for popular majors even surpassed those of several key academic senior high schools (Shanghai Vocational Education Online, 2024). In 2025, Guangzhou offered 40 admission places in the articulation program. The minimum admission cutoff was set at 627, the same as the third-tier cutoff for ordinary academic high schools (Guangzhou Municipal Education Bureau, 2025).

Despite growing policy attention, existing empirical research on the articulation program remains relatively limited and fragmented. A first strand examines program design and implementation, including training models, institutional arrangements, and curriculum articulation between secondary and undergraduate stages (L. Liu, 2023; Y.-L. Liu et al., 2015; Sun et al., 2016). A second strand focuses on student outcomes and quality assurance, addressing learning performance, the cultivation of vocational competencies, and the evaluation of training quality in pilot institutions (W. Liu et al., 2020). A third strand investigates the policy logic and governance dimension, analyzing the rationale, implementation dilemmas, and institutional safeguards associated with articulation policies at provincial and municipal levels (Xiong et al., 2022). In addition, some studies explore social recognition and public perceptions of the articulation route, including how stakeholders interpret its status relative to general academic pathways (Zhang et al., 2023; Nian, 2022). Research at the student level has largely concentrated on motivation, learning status, and program satisfaction among participants in articulation classes, often treating them as a relatively homogeneous group (Jiang et al., 2020).

However, high-scoring students, those whose examination scores would qualify them for admission to key academic senior high schools, have received little attention. Existing work has seldom examined why such students would voluntarily choose the articulation program, how they evaluate its risks and benefits, or how their decisions interact with the policy goal of promoting vocational–academic integration. To answer these questions, this study employs rational choice theory as an analytical framework and focuses on the articulation program as a strategic case through which to examine the decision-making logic underlying this educational choice.

## 2. Conceptual Framework

Alternative perspectives, including theories of educational and career decision-making, are also relevant to pathway choice. For example, recent reviews have highlighted the importance of approaches such as Career Construction Theory, Social Cognitive Career Theory, and Cognitive Information Processing Theory in explaining students’ educational and career decisions (Wang et al., 2024). Eccles-Parsons et al. (1983) argued that every task entails both benefits and costs; when the perceived costs of an option exceed its expected benefits, individuals tend to avoid it. Research on vocational and educational pathway choice has likewise shown that students’ perceptions of intrinsic value, utility value, and the feasibility of further progression can significantly shape their judgments (Antunes et al., 2026). However, RCT is particularly suitable here because this article is concerned not simply with career preference formation, but with educational pathway choice under conditions of institutional stratification, family involvement, credential competition, and the unequal social status of vocational education in China. Coleman’s framework offers a micro–macro analytical mechanism for understanding how purposive individual actions generate patterned social outcomes (Coleman, 1990).

At the same time, rationality in this study is not understood as perfect calculation under full information. Rather, it is approached in terms of bounded rationality. Rubinstein (1998) conceptualized bounded rationality as a procedural process of choice, in which decision-makers do not frictionlessly maximize given preferences, but instead rely on decision procedures that guide both what to do and how to decide. As de Clippel and Rozen (2024) further note, boundedly rational actors may face conflicting goals, rely on heuristics and rules of thumb, hold context-sensitive and unstable preferences, or settle for satisfactory rather than fully optimal outcomes. This perspective is relevant to educational choices made under uncertainty, unequal information, and institutional constraints. Rational choice research has shown that educational decisions are shaped not only by long-term utility, but also by expectations of success and the desire to reduce the risk of failure (Gabay-Egozi et al., 2010).

**Figure 1:**
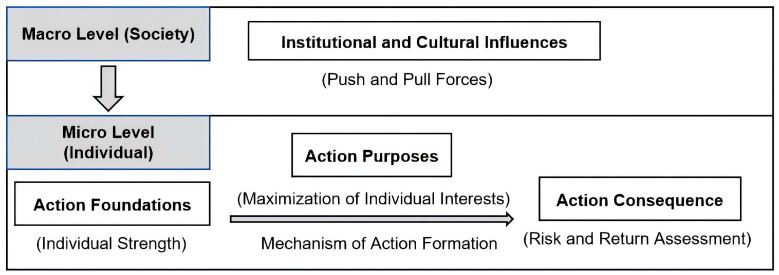
Analytical Framework of High-scoring Students’ Participation in the Articulation Program Based on Rational Choice Theory.

Based on rational choice theory and its established conceptual foundations, the factors influencing individual rational action, including actors, resources, interests, and rationality, can be grouped into four analytical dimensions: action foundations, action purposes, action consequences, and social institutional and cultural influences (Zhou & Xie, 2024). Specifically, individual competence constitutes the foundation of action; the pursuit of individual utility maximization represents the purpose of action; the actor’s value assessment of action outcomes, manifested in evaluations of risk and return, reflects the consequence of action. Meanwhile, social institutions and cultural norms at the macro-system level exert both direct and indirect influences on individual choices, forming a dynamic interplay between facilitating forces and constraining forces that either encourage or discourage participation in the articulation program (see Figure 1). This behavioral logic exemplifies the process through which micro-level conditions lead to micro-level outcomes, while these micro processes remain embedded within and influenced by macro-level structures.

## 3. Materials and Methods

### 3.1. Data Sources

To construct a broad and diverse corpus of public discourse, the study collected public discourse data from two primary sources: mainstream media reports and user-generated content on digital platforms, as shown in Table 1. Although the term is rendered in English as “Vocational Secondary–Undergraduate Articulation Program” for international readers, the Chinese term “Zhongben Guantong” is the official and most widely used expression in Chinese policy documents, media reports, admission information, and online discussions. This multi-source design was intended to capture not only institutionally framed narratives, but also more ambivalent and alternative evaluations, including debates over whether “Zhongben Guantong” should be understood as a “shortcut,” a “chicken rib,” a pressure-avoidance strategy, or a risky compromise.

The media search covered reports published between 1 April 2014 and 7 November 2024. The starting point reflects the year when Zhongben Guantong entered public visibility as a Shanghai-based articulation reform. Following Cheng and Qi (2024), this study used Tencent News as the main search entry for identifying relevant media texts, while also cross-checking against the original publishing sources. The initial search produced 111 items related to “Zhongben Guantong”. These items were first screened by title, source, and full-text relevance. Duplicate reposts, brief admission notices, purely promotional materials, and items with only marginal relevance to students’ educational choices were excluded. The final media corpus included 12 focal texts, totaling approximately 40,000 Chinese characters. The selected media texts were categorized by source type and regional focus. The corpus included local government-affiliated mainstream media, national state-authorized mainstream media, commercially oriented feature journalism, and vocational education sector information sources. Given the Shanghai-based origins and early institutional development of Zhongben Guantong, the media corpus was Shanghai-centered while several reports also extended to the provinces of Zhejiang and Jiangsu in the Yangtze River Delta region. Existing studies indicate that the Yangtze River Delta is one of China’s most favorable regions for vocational education reform and regional integration (Zeng, 2021). Accordingly, the corpus should be understood as Shanghai-centered and Yangtze River Delta-related. Regional focus was used to clarify the contextual boundaries of the data.

Online discourse was collected through digital ethnographic observation on platforms with high engagement among parents and students, including Xiaohongshu and Zhihu. The data collection period ended on 7 November 2024. On Zhihu, discussions under the topic “Zhongben Guantong” reached 6.78 million views and 5930 comments. Using the GooSeeker crawler, 198 Xiaohongshu posts were collected; after screening for relevance and interaction intensity, four high-engagement posts and 923 associated comments were selected. Two high-traffic Zhihu threads were also sampled, producing 36 replies and roughly 20,000 words of text. The sampling strategy prioritized relevance, visibility, interaction intensity, and thematic richness. Media texts were labeled using the prefix MT (Media Text), followed by platform abbreviations (e.g., MT-PP for The Paper). Online discourse was labeled WL (Web-based Language) and similarly organized by platform (e.g., WL-XHS-1 for the first Xiaohongshu post).

### 3.2. Method

Thematic analysis (TA) is now widely used in qualitative research. Recent methodological scholarship has stressed that TA should not be regarded as a single, standardized analytic technique, but rather as a family of methods that differ in their philosophical assumptions, analytic procedures, and conceptualisations of themes (Braun & Clarke, 2022). In contrast to purely inductive forms of thematic analysis, theoretical thematic analysis refers to an approach to thematic analysis in which researchers draw on an established theoretical framework, research perspective, or predefined analytical dimensions as the point of departure, and employ a top-down strategy to code qualitative data, identify themes, and interpret meanings (Braun & Clarke, 2006). Rational choice theory provided the initial analytical framework, while close engagement with the empirical materials allowed the coding scheme and themes to be continuously revised and refined.

All discourse materials were imported into NVivo 15.0 for systematic coding. Deductively, four top-level analytical dimensions were constructed around action foundations, action purposes, action consequences, and institutional–cultural influences. Inductive coding was used to refine initial codes and subthemes within these predefined analytical dimensions. For example, initial codes such as “academic performance” and “personal traits” were identified through repeated engagement with the data and further compared during later stages of analysis. Table 2 provides illustrative examples of this coding process. Second-cycle coding was conducted to compare, merge, and reorganize the initial codes into more analytical subthemes. For instance, codes such as “academic performance” and “personal traits” were integrated into the subtheme of “individual competence”. The coding system’s saturation was carefully tested. As the number of analyzed texts increased, the emergence of new conceptual categories gradually declined. By the time the 14th text was coded, the coding outcomes had stabilized. Considering the potential volatility of saturation, an additional four interactive discourse samples were coded. The extracted conceptual elements showed a high degree of overlap with the existing coding scheme, and no new categories emerged. Potentially identifying personal details were removed, minimized, or replaced with generic descriptors where appropriate.

## 4. Results

### 4.1. Action Foundations: Individual Competence and Rational Family Support

At the individual level, action foundations are rooted not only in students’ relatively strong academic performance, but also in their perceived competence, learning preferences, and intrinsic interest. Some explicitly frame themselves as better suited to hands-on or application-based curricula rather than purely exam-driven study:

“My score could have secured admission to a top district-level high school in Shanghai, but I’m stronger in practice-based courses and have clear subject preferences”.

(MT-PP-7)

In terms of learning style and motivation, many students display a strong orientation toward autonomous, experientially grounded learning, rejecting the image of themselves as “caged birds” in a rigid academic system (MT-HZ-10) and emphasizing that theory becomes meaningful only when connected to practice (MT-SG-2). This orientation can be understood in terms of intrinsic value, or interest value, which refers to the anticipated enjoyment individuals expect from engaging in a task as well as the enjoyment actually experienced during task engagement (Eccles & Wigfield, 2020). Their professional aspirations tend to be closely aligned with personal interests: they frequently describe having “entered the major I truly desired” or “found a field I genuinely enjoy exploring” (MT-SG-9; MT-SH-12). By contrast, students who entered the program “just for the credential” without genuine interest often report regret and disengagement (MT-PP-7).

At the familial level, families that support the articulation program are typically portrayed as well-informed and pragmatically rational. Rather than uncritically adhering to the dominant “academic-first” norm, they make deliberate evaluations of their children’s academic strengths, interests, and psychological resilience, and judge which pathway is more sustainable. These families also tend to hold less stigmatized views of vocational schools, often recognizing the value of school culture, teacher commitment, and campus environment (MT-SL-3; MT-WH-4). In addition, they place considerable emphasis on the fit between program characteristics and children’s dispositions, such as choosing transportation-related majors for those interested in sports or hands-on mechanical activities (MT-HZ-10). They also take into account financial constraints and opportunity costs, acknowledging that the tuition-free vocational phase can ease economic pressure, while prolonged competition in the general academic track may impose substantial financial and emotional burdens (MT-SL-3; MT-WH-11).

### 4.2. Action Purposes: Individual Utility Maximization in Education and Career

The second dimension concerns action purposes, especially educational security and career development. Research in behavioral science indicates that most individuals are risk-averse (Dohmen & Falk, 2011). In the educational domain, the articulation program is widely perceived as a relatively secure route to a bachelor’s degree. Through the “3 + 4” model, students bypass the high-stakes uncertainty of the National College Entrance Examination (NCEE) and instead progress via a transition examination that is considered more controllable:

“Even key district-level high schools are no longer a guaranteed route to university. It’s better to ‘lock in’ a university early. After all, you still earn the same bachelor’s diploma and degree”.

(MT-SG-9)

“The most important thing about ‘Zhongben Guantong’ is earning a bachelor’s degree”.

(WL-ZH-1)

For many middle-class families, the bachelor’s credential is framed as a “non-negotiable necessity” (MT-SL-3), reinforced by rising credential thresholds in the labor market, for example, public kindergartens in some cities now routinely require bachelor’s degrees (MT-SL-3). In this context, the articulation program’s high transition rate and reduced exam pressure are repeatedly highlighted. Students emphasize that they “don’t get pushed like high school students” and that, in some cases, “39 out of 40 classmates passed the transition exam” (WL-ZH-2). At the same time, this decision is accompanied by emotional ambivalence. Families acknowledge the symbolic loss involved in giving up an offer from a key high school and worry about “seven years of all-in commitment” (MT-SG-9). Yet this hesitation is frequently outweighed by the perceived uncertainty of the conventional academic route: “We don’t know what the result will be, no matter how much we invest” (MT-HZ-10). Under such conditions, the articulation program is often judged to be the more rational gamble.

Utility maximization is also evident in relation to career development. The program is described as offering distinct labor-market advantages. School administrators and media accounts suggest that articulation graduates sometimes achieve better employment outcomes than peers who enter universities through NCEE:

“Based on employment outcomes of the first three cohorts of ‘Zhongben Guantong’ graduates, their job performance and placement rates are slightly higher than those of comparable students admitted to universities through NCEE”.

(MT-WH-11)

These advantages are often attributed to students’ earlier and more sustained exposure to vocational training. Articulation students are characterized as having more standardized technical skills, stronger operational proficiency, and higher professional identification due to their three-year vocational training (MT-WH-4; MT-WH-6). In certain regions, teacher-education articulation programs even offer directed employment and guaranteed establishment in the local school system, making them especially attractive in a labor market where permanent teaching posts are hard to obtain (MT-GM-8).

### 4.3. Action Consequence: Risk–Benefit Evaluation—An Appealing Shortcut Despite Its Limitations

High-scoring students’ decisions to enter the articulation program emerge from a boundedly rational evaluation of anticipated risks. They may perceive the academic track as excessively costly and therefore choose the articulation route as a lower-risk alternative.

Discourse participants repeatedly acknowledged structural concerns, such as the relatively modest institutional status of articulation universities and the limited flexibility inherent in early specialization. As one commentator noted, “A degree from a second-tier articulation university feels somewhat limiting, the platform isn’t strong enough” (WL-ZH-2), while another student worried that “starting in an engineering major at 15 makes your thinking rigid” (WL-ZH-2). Others pointed to uncertainties in academic progression and institutional variation, observing that “students who fail to meet required credits may only receive a certificate of completion rather than a degree” (MT-WH-1), and that the learning atmosphere in some schools remains insufficiently motivating: “Most people just drift along, the overall academic climate is weak” (WL-ZH-2). The articulation model faces practical barriers related to academic preparation and labor-market recognition. “At a certain applied technology university, articulation students’ average scores in foundational courses such as advanced mathematics were significantly lower than those of students in parallel classes” (MT-PP-7). Meanwhile, information asymmetry and limited employer recognition further constrain graduates’ career prospects. “My ‘Zhongben Guantong’ background could not even get me an interview for a factory worker position, because they only considered graduates from regular universities” (WL-ZH-1).

Yet these concerns coexist with an equally strong recognition of the pathway’s perceived advantages, especially in comparison with the pressure associated with the conventional academic route. Academic pressure has long been identified as a major impediment to students’ academic success (Shankar & Park, 2016). Sustained academic stress is also regarded as a key antecedent of academic burnout, which may in turn lead to emotional exhaustion, cynicism, and diminished self-confidence (Jung et al., 2015). Across news reports and online discussions, students and families repeatedly emphasized the psychological relief and stability afforded by bypassing NCEE, with one parent remarking that “without the huge pressure of NCEE, the whole family feels more relaxed” (MT-SG-9). Students often reference peers who experienced severe stress or even mental health crises in elite academic tracks, and they consciously seek to avoid similar trajectories:

“Studying all day just to fight for a bachelor’s degree feels meaningless, ‘Zhongben Guantong’ offers another route to the same goal” (MT-HZ-10); “A friend entered a top high school but developed depression in Grade 11 and eventually dropped out”.

(MT-SL-3)

The articulation program was also frequently described as cost-effective, enabling students to obtain a bachelor’s degree while benefiting from tuition-free vocational schooling. Participants further highlighted the developmental value of sustained practical training, noting that articulation students often possess “more standardized technical skills and stronger professional awareness” (MT-WH-4). Many framed their trajectories in terms of incremental confidence-building, as reflected in a student’s comment:

“Compared with students admitted through NCEE, her stronger professional foundation gave her a clear advantage upon entering university. Over three years, I earned several certificates which make me feel more confident as I begin university”.

(MT-PP-7)

Taken together, these accounts suggest that the articulation program is not valued because it is seen as risk-free, but because its risks are often judged to be more manageable, more predictable, or more worthwhile than those associated with the conventional academic route.

### 4.4. Institutional and Cultural Influences: Push–Pull Dynamics in a Stratified System

At the macro-system level, social institutions and cultural norms exert profound influence on individual actors’ decision-making and behavioral orientations. These macro-level forces operate through a dynamic interplay of push and pull factors, each producing either facilitating or constraining effects on individual choices.

On the one hand, the push forces motivating high-scoring students to voluntarily choose the articulation program stem from the combined influences of industrial demand and policy support. China’s economic restructuring has reshaped the definition of technical and skilled labor. The labor market is transitioning from a traditional structure dominated by “low-skill, quantity-oriented” workers to one characterized by “high-skill, quality-oriented” talent (Bai, 2017). Yet the internal composition of the workforce remains imbalanced, with a persistent shortage of high-level skilled professionals. As one report noted,

“In Shanghai, the proportion of skilled workers at the primary, intermediate, and advanced levels is 39:42:19, which still lags far behind the 15:50:35 ratio found in developed countries”.

(MT-WH-1)

To address this disparity, the articulation program serves as an educational response to industrial upgrading, aiming to produce high-quality, application-oriented technical talent at the undergraduate level. “Compared with traditional undergraduate programs, “Zhongben Guantong” education is characterized by its technological orientation. It targets the cultivation of high-level applied professionals, combining elements of engineering and technical education, with an emphasis on the latter” (MT-WH-1). The articulation model’s policy-driven nature further strengthens its institutional legitimacy. As one document emphasized, “This model is highly policy-oriented, typically implemented in specific regions, industries, and sectors, to cultivate urgently needed professionals and alleviate local talent shortages” (MT-GM-8).

On the other hand, the resistance forces that constrain high-scoring students’ participation in the articulation program arise from a combination of deep-seated cultural beliefs. For a long time, within the context of East Asian Confucian culture, vocational education has been positioned as a “second-tier” form of education, facing persistent problems such as weak attractiveness and low social recognition (Guan & Blair, 2024).

“In our generation’s view, students in secondary vocational or technical schools are generally of lower quality. High school is a crucial stage. Will the school care about the child’s wellbeing? Can they manage themselves? What kind of classmates will they have?”.

(MT-HZ-10)

Such stereotypes reinforce societal resistance and aversion toward vocational education. The case of one student and her mother vividly illustrates this tension. They faced strong opposition from teachers and relatives when choosing a vocational school. The homeroom teacher even stated, “Those schools are for students ranked below the top 500, you absolutely shouldn’t go there” (MT-PP-7). This kind of dismissive labeling deepens the social stigmatization of vocational education and reinforces entrenched hierarchies within China’s educational system. As one analysis observed, “The vast majority of junior high students and their parents still regard NCEE as the first choice, while the articulation model remains a secondary option” (MT-GM-8).

## 5. Discussion

What the corpus can show, however, is how high-scoring students’ participation in the articulation program is publicly represented, explained, and justified in a specific regional and institutional context. Educational choice among high-scoring students in China is deeply embedded in a highly stratified and examination-driven system, where academic success is closely tied to family expectations, future mobility, and social recognition. Against this backdrop, choosing the articulation program should not be interpreted as a deviation from achievement-oriented norms, but rather as a rational response to uncertainty, pressure, and the desire to secure a more predictable route to higher education. These accounts construct the pathway as a strategy for balancing several concerns at once: securing a bachelor’s degree, reducing exposure to high-stakes academic competition, preserving psychological sustainability, and gaining vocationally oriented advantages. In this sense, the findings do not establish students’ actual motivations in a causal sense; rather, they reveal the interpretive repertoire through which students, families, media actors, and online commentators make this choice publicly intelligible.

This finding contributes to comparative discussions on educational choice and the attractiveness of vocational education. When strong academic competition, permeability to higher education, though the unequal status of vocational routes coexist, even high-achieving students may come to regard vocationally oriented pathways as rational and strategically advantageous choices. In China, education is closely intertwined with social status and an individual’s position in the social hierarchy (P. Wu et al., 2025). Thus, even when students pursue a vocationally oriented pathway, their decisions remain anchored in a cultural environment where the bachelor’s degree functions as a dominant marker of educational legitimacy. It resonates with research suggesting that the attractiveness of VET is not fixed, but depends on how students and families evaluate status, security, and future opportunities under specific institutional conditions (Pilz, 2025).

The findings also suggest that the legitimacy of vocationally oriented pathways is not produced solely by formal policy design. It is also shaped through public narratives that negotiate the tension between policy incentives and persistent cultural hierarchies. In the corpus, positive accounts often emphasize educational security, practical training, reduced examination pressure, and alignment with industrial demand. At the same time, ambivalent and critical accounts highlight concerns about institutional status, early specialization, limited flexibility, employer recognition, and the symbolic loss associated with not entering the academic high-school route. This tension also shows that educational rationality in this context cannot be reduced to a purely individual calculation of utility. Rather, it is bounded, relational, and status-sensitive. Students and families make decisions under uncertainty while also responding to family expectations, mental-health concerns, credential pressures, and the symbolic hierarchy between academic and vocational education.

### 5.1. Practical Implications

Because the analyzed materials present families as evaluating the articulation program under uncertainty, policy and school responses should address not only institutional design, but also the informational and symbolic conditions of choice. This suggests that policymakers and schools should not treat the articulation program merely as a technical arrangement for expanding vocational–academic permeability. They also need to address the informational and symbolic conditions under which families evaluate the articulation program. Clearer information is needed about transition requirements, undergraduate destinations, degree recognition, employment outcomes, and possibilities for later mobility. At the same time, schools should provide guidance that helps families assess whether the articulation program fits students’ interests, learning styles, academic foundations, and long-term development, rather than presenting it simply as a “shortcut” to a bachelor’s degree. Public communication should also avoid both over-promoting the articulation program and reproducing stigmatizing representations of vocational education. Prior research suggests that students are more likely to make confident and sustainable choices when parents hold realistic expectations and communicate encouragement (Trinidad, 2019), and when young people are able to connect schooling with plausible future selves (Efthymiadou et al., 2025). Comparative research on German vocational schools also suggests that transition pathways become more effective when institutional support, information, and guidance are systematically provided (Dörffer & Bernhard, 2025). The corpus suggests that public confidence depends not only on policy design, but also on whether students and families can regard the articulation program as educationally legitimate, socially respectable, and developmentally sustainable.

### 5.2. Limitations and Future Directions

Several limitations should be acknowledged. First, this study draws on qualitative discourse data from media reports and online interactions. Much of the discourse analyzed here was produced after educational choices had already been made, which means that participants’ accounts may involve retrospective interpretation, selective recall, or post hoc rationalization. Second, these materials represent only those individuals who are willing and able to express their views in public or online spaces. As such, the findings may not fully capture the perspectives of students from less visible backgrounds or from schools with weaker program capacity. Third, online discourse consists of posted and publicly available expressions that may have been edited, filtered, or strategically presented, and thus may not fully reflect participants’ immediate or unmediated views. Fourth, because the analysis focuses on expressed perceptions rather than observed long-term outcomes, the study cannot support strong causal claims regarding graduates’ later academic, occupational, or identity trajectories. Future research could address these limitations through interviews, surveys, or mixed-method designs, and by using longitudinal approaches to examine how students’ academic performance, career outcomes, and identity formation develop across the seven-year articulation program. In particular, the present retrospective analysis may serve as a basis for future quantitative and longitudinal research on potential applicants to the articulation program. Comparative research across provinces or articulation models would also help clarify regional variation in policy implementation and social recognition. In addition, further research is needed to examine the role of digital culture and online discourse in shaping youth perceptions of educational choice in contemporary China.

## 6. Conclusions

This study developed a micro-macro analytical framework, grounded in rational choice theory, to interpret why high-scoring students increasingly choose the articulation program. The findings show that public accounts of these choices are organized around three interconnected mechanisms of action formation: action foundations rooted in individual competence and rational family reasoning; action purposes centered on maximizing utility through educational security and career-oriented development, and action consequences evaluated through risk–benefit assessments that frame the articulation model as a comparatively efficient and psychologically sustainable pathway. Across these mechanisms, institutional and cultural dynamics function simultaneously as enabling and constraining forces. The articulation model’s policy-driven expansion and alignment with industrial demand generate structural “push” forces, while persistent credential hierarchies and vocational stigma exert cultural “pull” or resistance forces. In the analyzed discourse, these choices are made intelligible as emerging at the intersection of these competing pressures.

Overall, the study shows that high-scoring students’ participation in the articulation program is not a rejection of academic norms but a reconfiguration of them. It reflects a strategic re-calibration of educational pathways within a system where uncertainty, competition, and credential expectations continue to structure individual aspirations and opportunities. The articulation program’s growing attractiveness thus signals both the potential for diversified educational routes and the ongoing importance of addressing entrenched cultural inequalities in the valuation of vocational education.

## Figures and Tables

**Table 1:** Basic Information on Collected Discourse Materials.

Code	Platform Category	Text Title	Date	Regional Focus	Source
MT-WH-1	Local government-affiliated mainstream media	Exploring the Path of Elite Training in Vocational Education	25 April 2014	Shanghai	Wenhui Daily
MT-SG-2	Local government-affiliated mainstream media	With a Score Meeting the Cutoff for Key High Schools, Why Did She Choose the “Secondary-Undergraduate Articulation” Track?	8 December 2020	Shanghai	Shanghai Observer
MT-SL-3	Commercially oriented feature journalism	Why Do High-Scoring Students Choose Vocational Schools?	22 July 2021	Zhejiang	Sanlian Lifeweek
MT-WH-4	Local government-affiliated mainstream media	Scoring 703 on the High School Entrance Exam, He Chose Vocational School—Vocational Education Is No Longer a Last Resort but a New Path in Life	10 August 2021	Shanghai	Wenhui Daily
MT-WH-5	Local government-affiliated mainstream media	She Scored 701.5 and Ranked Top 10 in Her Grade, Yet Chose the Articulation Program Instead of a Top Academic High School: “The Best Choice Is the One That Fits You”	16 August 2022	Shanghai	Wenhui Daily
MT-WH-6	Local government-affiliated mainstream media	Shanghai Has Established 65 Articulation Majors—Seven-Year “Long-Cycle” Training, Employment Outcomes Viewed as Highly Favorable	1 February 2023	Shanghai	Wenhui Daily
MT-PP-7	Commercially oriented feature journalism	Shortcut or “Chicken Rib”? Multiple Provinces Step Up Exploration of Secondary-to-Bachelor “Direct Promotion”	19 May 2023	Shanghai Zhejiang	The Paper
MT-GM-8	National state-authorized mainstream media	Why Is the Vocational Education Articulation Model So Widely Welcomed?	29 August 2023	Shanghai Zhejiang Jiangsu	Guangming Daily
MT-SG-9	Local government-affiliated mainstream media	Sprinting Toward the High School Entrance Exam: Experts Debate Whether the Articulation Model Should Expand, While Parents Struggle Over Whether to “Lock In” a University Early	8 May 2024	Shanghai	Shanghai Observer
MT-HZ-10	Local government-affiliated mainstream media	Why Did Children Scoring Over 600 Choose the Integrated Secondary-Undergraduate Track? Behind Three Families’ Decisions Lies Self-Reconciliation and Parental Understanding	17 July 2024	Hangzhou	Hangzhou Daily
MT-WH-11	Local government-affiliated mainstream media	Why Do High-Scoring Students Choose the Articulation Model?	21 July 2024	Shanghai	Wenhui Daily
MT-SH-12	vocational education-sector information sources	Why Are More High-Scoring Students Choosing Vocational Colleges?	21 August 2024	Shanghai	Shanghai Vocation Education Online
Online Interactive Discourses					
WL-ZH-1	User-generated online platform	Should You Enroll in the Secondary–Undergraduate Articulation Program?	29 March 2018	Shanghai	Zhihu
WL-ZH-2	User-generated online platform	Is the Articulation Model in Shanghai Actually Good?	19 June 2020	Shanghai	Zhihu
WL-XHS-1	User-generated online platform	Seven Years Ago, I Turned Down a Key High School for the Articulation Path—Do I Regret It?	30 September 2024	Shanghai	Xiaohongshu
WL-XHS-2	User-generated online platform	An Ordinary Student in Shanghai Entered the Articulation Program—No More Involution	15 July 2024	Shanghai	Xiaohongshu
WL-XHS-3	User-generated online platform	A Forward-Looking Decision Made Seven Years Ago: Taking the Shortcut Through the Articulation Program	10 February 2023	Shanghai	Xiaohongshu
WL-XHS-4	User-generated online platform	Key High School or Articulation Program?	3 December 2023	Shanghai	Xiaohongshu

**Table 2:** Illustrative Coding Examples for the Analytical Dimension of Individual Strengths.

Dimension	Subtheme	Initial Code	Raw Data	Number
Individual strengths	Individual competence	Academic performance	MT-GM-8: Some students scored well above the admission line for elite academic high schools but still chose the integrated pathway.	26
		Personal traits	MT-PP-7: Although she was eligible for a key high school, her subject imbalance and preference for practice-oriented learning made this pathway more suitable.	5
		Learning attitude	MT-WH-11: Students need to prepare major-related knowledge in advance and keep up with all subjects; this pathway is not seen as a shortcut.	10
		Career orientation	MT-WH-11: Choosing vocational education first was seen as a way to turn personal interests into a future engineering career through stronger practical training.	10
	Family educational values	Family educational beliefs	MT-WH-5: Families believed that choosing a path aligned with the child’s interests and development was more beneficial during adolescence.	17
		Family economic conditions	MT-SL-3: Due to financial constraints, the family sought an alternative pathway that would better suit their daughter’s future.	3
		Family educational decision- making	MT-WH-11: After comparing the pros and cons of academic high schools and the integrated pathway, both parents and child chose the latter.	15

## Data Availability

The raw data supporting the conclusions of this article will be made available by the authors on request.
